# Myeloid-derived suppressor cells: key immunosuppressive regulators and therapeutic targets in hematological malignancies

**DOI:** 10.1186/s40364-023-00475-8

**Published:** 2023-03-29

**Authors:** Shifen Wang, Xingyun Zhao, Siwen Wu, Dawei Cui, Zhenshu Xu

**Affiliations:** 1 Department of Blood Transfusion, The First Affiliated Hospital, Zhejiang University School of Medicine, Hangzhou, China; 2 Department of Hematology, Fujian Institute of Hematology, Fujian Provincial Key Laboratory on Hematology, Fujian Medical University Union Hospital, Fuzhou, China

**Keywords:** Hematological malignancies, Myeloid-derived suppressor cells, Immunosuppressive regulator, Tumor microenvironment, Immunotherapy

## Abstract

The immunosuppressive tumor microenvironment (TME) supports the development of tumors and limits tumor immunotherapy, including hematological malignancies. Hematological malignancies remain a major public health issue with high morbidity and mortality worldwide. As an important component of immunosuppressive regulators, the phenotypic characteristics and prognostic value of myeloid-derived suppressor cells (MDSCs) have received much attention. A variety of MDSC-targeting therapeutic approaches have produced encouraging outcomes. However, the use of various MDSC-targeted treatment strategies in hematologic malignancies is still difficult due to the heterogeneity of hematologic malignancies and the complexity of the immune system. In this review, we summarize the biological functions of MDSCs and further provide a summary of the phenotypes and suppressive mechanisms of MDSC populations expanded in various types of hematological malignancy contexts. Moreover, we discussed the clinical correlation between MDSCs and the diagnosis of malignant hematological disease, as well as the drugs targeting MDSCs, and focused on summarizing the therapeutic strategies in combination with other immunotherapies, such as various immune checkpoint inhibitors (ICIs), that are under active investigation. We highlight the new direction of targeting MDSCs to improve the therapeutic efficacy of tumors.

## Introduction

Myeloid-derived suppressor cells (MDSCs) are an immature and heterogeneous cell population that can inhibit T-cell function. In the 1970s, a population of immunosuppressive myeloid cells with suppression of T-cell function in tumor-bearing mice was accidentally discovered [1–3]. These cells were called “immature myeloid cells” or “myeloid suppressor cells”. Over the next decades, various studies have shown their ability to suppress T-cell activation and function and their production from immature bone marrow cells, and the MDSC definition was proposed in 2007 [4]. In pathological conditions such as cancer, infection, chronic inflammation, trauma, bone marrow transplantation, sepsis, and autoimmune diseases, the numbers of MDSCs are massively amplified and aggregated to the lesions, where they are involved in immune escape, immune tolerance, inflammatory response, and other processes [5]. Recent studies have found that MDSCs are involved in pregnancy and neonatal biological effects and have been noted in COVID-19 patients [6–9].

The development of hematological malignancies depends on the evolution of cancer cells and the ecosystem in which they grow, and the immune microenvironment has become an area of intense research. As a component of the tumor immune microenvironment, MDSCs and their related factors and functions, such as reactive oxygen species (ROS), indoleamine 2,3-dioxygenase (IDO), arginase 1 (ARG1), adenosine, and negative immune checkpoints, can remodel the suppressive immune microenvironment of tumors [10]. MDSCs may exert inhibitory effects on immune responses by modulating the function of NK cells and CD4^+^ and CD8^+^ T cells, leading to an imbalance in the immune system and immune editing of tumors into an immune escape phase [10]. However, this also makes MDSCs a potential target with far-reaching therapeutic treatments to overcome the immunosuppressive microenvironment of tumors. In recent years, progress has been made with several exploratory therapies that target MDSCs for the treatment of hematological malignancies. However, many questions regarding the mechanisms of MDSC activation, differentiation, and function remain unanswered. In this review, we summarize existing findings on the role and function of MDSCs in various hematological tumors and discuss treatments targeting MDSCs to provide ideas for future research and therapies.

## Phenotypic characteristics and functions of MDSCs

**Fig. 1:**
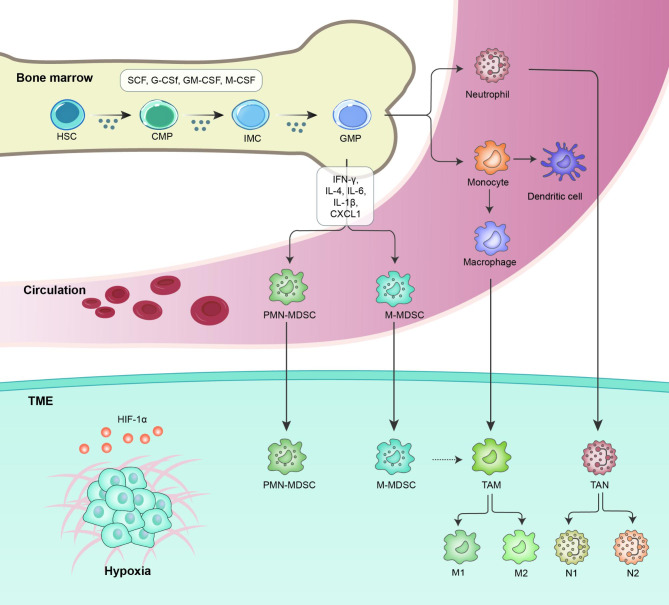
**Stages of MDSC differentiation and accumulation.** Hematopoietic stem cells (HSCs) differentiate in bone marrow into common myeloid progenitors (CMPs), which can further differentiate through the hematopoietic system. Under physiological conditions, CMPs can differentiate into neutrophils or monocytes and subsequently into MΦs or DCs. However, under pathologic conditions, immature myeloid cells (IMCs) are expanded and converted to immunosuppressive MDSCs, which include monocytic myeloid-derived suppressor cells (M-MDSCs) and polymorphonuclear myeloid-derived suppressor cells (PMN-MDSCs). In tumors, M-MDSCs can further differentiate into tumor-associated macrophages (TAMs) (M1 and M2 phenotypes). Different cytokines are involved in the whole differentiation process. Growth factors such as SCF, G-CSF, GM-CSF, and M-CSF regulate myelopoiesis progression, inducing the expansion of MDSCs. In the presence of proinflammatory cytokines such as IFN-γ, IL-4, IL-6, IL-1β, and CXCL1, IMCs are pathologically activated and then differentiate into M-MDSCs and PMN-MDSCs. Hypoxia in the TME facilitates the expression of hypoxia-inducible factor 1-alpha (HIF-1α), which leads to MDSC recruitment and accumulation. TME, tumor microenvironment

The varied population of myeloid cells originating from hematopoietic stem cells (HSCs) is composed primarily of monocytes and granulocytes. Monocytes convert into macrophages (MΦs) and dendritic cells (DCs) in tissues, where they help maintain homeostasis and respond to inflammatory conditions, while granulocytes terminally differentiate into polymorphonuclear neutrophils, basophils, and eosinophils [11]. In healthy individuals, pathogen-associated molecular patterns (PAMPs) released by various pathogens, and damage-associated molecular patterns (DAMPs) usually released by damaged tissues or cells, that act as danger signals, which drive myeloid cells to differentiate into the above mentioned mature cells, triggering innate and adaptive immune responses [7]. At this time, in the process of defending against pathogens, the defense by myeloid and lymphoid reactions needs to maintain a balance (Fig. 1) [6]. In contrast, under certain pathological conditions, particularly cancer and chronic inflammation, persistent aberrant signals stimulate myelopoiesis, which allows immature myeloid cells to stagnate during differentiation and acquire immunosuppressive properties. These cells are known as MDSCs (Fig. 1) [7].

Based on their origin and characteristics, murine and human MDSCs are subdivided into two major subgroups: monocytic MDSCs (M-MDSCs) and polymorphonuclear/granulocytic MDSCs (PMN-MDSCs, also known as G-MDSCs) [8]. In most cases, PMN-MDSCs account for more than 70% of all MDSCs, while M-MDSCs account for less than 30% [11, 12]. A third small subgroup of MDSCs present in tumors has been reported to be effective in suppressing T cells in vivo and in vitro, significantly promoting tumor growth and metastasis [9, 13, 14]. These cells are called early MDSCs (e-MDSCs). Furthermore, studies in umbilical cord blood (UCB) or metastatic pediatric patients with sarcoma in the peripheral blood have identified another MDSC subgroup, named fibrocytic MDSCs (F-MDSCs) [15, 16]. A subset of myeloid precursor cells with the MDSC phenotype but no immune function, designated MDSC-like cells (MDSC-LCs), are detectable in the early phases of tumor progression [17]. According to current publications, precursor cells that differentiate into PMN-MDSCs can also emanate from the monocyte lineage and are termed monocyte-like precursors of granulocytes (MLPGs) [18]. The proliferation of MLPG is regulated by the downregulation of the retinoblastoma gene (Rb1). The most notable characteristic of MDSCs is that they are present in extremely low numbers in the peripheral blood of healthy people but are significantly increased in an inflammatory or infectious or neoplastic state. PMN-MDSCs are morphologically and phenotypically similar to neutrophils, whereas M-MDSCs resemble monocytes, which is consistent with the origin of these cells [17, 19]. In recent years, many studies have found that PMN-MDSCs have different gene profiles from neutrophils [20, 21], and proteome profiling studies of MDSCs have determined that these cells constitute a myeloid cell population with unique characteristics and protein functions [22, 23]. Human PMN-MDSCs have the morphology of immature to mature polymorphic nuclei neutrophils [13, 24, 25]. In vitro studies with mature neutrophils confirmed their ability to transform into cells with immunosuppressive functions under specific conditions [26, 27]. With decreased HIF-1 and reduced STAT3 activity in the hypoxic tumor microenvironment (TME), M-MDSCs acquire the phenotype of tumor-associated macrophages (TAMs) [28, 29]. According to a recent study, M-MDSCs in tumor-bearing mice can transition into PMN-MDSCs [30]. This phenotypic alteration was triggered by transcriptional silencing of Rb1 through epigenetic modifications by histone deacetylase 2 (HDAC-2) [30]. Interestingly, not only can MDSCs continue to further differentiate into DCs, MΦs, and TAMs [28–31], but factors or pathways that mediate MDSC recruitment can also cause mature myeloid cells to differentiate into MDSCs. For instance, CD14 + monocytes are able to develop into M-MDSCs via interleukin (IL)-10 and PGE2 in the TME [32, 33], and neutrophils can transform into PMN-MDSCs expressing LOX-1 through endoplasmic reticulum (ER) stress [20]. The notion that MDSCs are the origin of pathologically activated neutrophils and monocytes is supported by these studies, and these MDSCs have unique genomic, proteomic, and metabolic characteristics [8]. There is still controversy about whether MDSCs are also the pathological state of mature myeloid cells [34].

Mouse cells that coexpress the myeloid antigens GR-1 and CD11b (GR-1^+^CD11b^+^) are referred to as MDSCs [35]. Different levels of Ly6G and Ly6C expression and two subtypes of the granulocyte marker Gr-l permit further classification of MDSCs as PMN-MDSC (CD11b^+^LY6G^+^LY6C^low^) and M-MDSC (CD11b^+^LY6G^−^LY6C^high^) subtypes [36]. However, human leukocytes do not express Gr-1. In human peripheral blood mononuclear cells (PBMCs), M-MDSCs and G-MDSCs express the CD11b^+^CD14^+^CD15^−^CD33^+^HLA-DR^low/−^ and CD11b^+^CD14^−^CD15^+^HLA-DR^low^CD66b^+^ designations, respectively [17]. The MDSC subgroup can also be recognized by other phenotypic molecules, such as CD84 and junctional adhesion molecule-like protein (JAML) [8, 37]. However, the CD11b^+^CD14^+^CD15^−^CD33^+^HLA-DR^low/−^ phenotype of M-MDSCs and the CD11b^+^CD14^−^CD15^+^HLA-DR^low^CD66b^+^ phenotype of G-MDSCs are also monocyte and granulocyte phenotypes, respectively. In human, although PMN-MDSCs appear in the low-density interphase (1.077 g/ml) after density gradient centrifugation, whereas neutrophils appear in the high-density interphase (1.1–1.2 g/ml), both gradient components can pass through a gradient contaminated with the other [17]. Conversely, due to the expression of MHC class II molecules, which are only expressed on monocytes (HLA-DR^+^), human M-MDSCs may be easily identified from monocytes [14]. In mice, Ly6G and Ly6C expression by myeloid cells appears to be variable based on inflammatory stimuli. Jia, et al. found that CD48 can distinguish between PMN-MDSCs and M-MDSCs in a CD11b^+^Ly6G^low^Ly6C^high^ cell sepsis model with phenotypes of both [38]. Determining the cell-surface markers and gating strategies that specifically identify the various populations of MDSCs is a core focus of continued studies. Since the MDSC phenotype is not exclusive, it is inappropriate to define MDSCs solely by concentrating on immune cell markers. Identifying MDSCs also requires determining whether they have immunosuppressive properties. The phenotypic and immunosuppressive features of MDSCs in hematological malignancies are shown in Table 1.

**Table 1:** Phenotypes and immunosuppressive feature of MDSCs in hematological malignancies

Diseases	MDSCs type	Phenotype	Immunosuppressive features	Clinical significance	Year/reference
DLBCL	↑M-MDSCs	CD45^+^CD11b^+^CD33^+^HLA-DR^low/−^CD14^+^CD15^−^	/	Blood concentration of MDSCs and Treg cells may be good prognostic markers for overall survival after 2 years in R/R DLBCL.	2021 [59]
	↑PMN-MDSCs	CD45^+^CD11b^+^CD33^+^HLA-DR^low/−^CD14^−^CD15^+^			
DLBCL	↑M-MDSCs	CD14^+^HLA-DR^−^	↑IL-10, S100A12, PD-L1	Only M-MDSCs number was correlated with the International Prognostic Index, event-free survival, and number of circulating Tregs.	2016 [55]
	↑PMN-MDSCs	Lin^−^CD123^−^HLA-DR^−^CD33^+^CD11b^+^			
DLBCL	↑M-MDSCs	CD14^+^HLA-DR^−/low^	↑IL-35	Increased levels of M-MDSCs are positively associated with tumor progression and inversely correlated with OS. The level of M-MDSCs can be defined as a biomarker for a poor prognosis in DLBCL patients.	2021 [58]
B-NHL	↑M-MDSCs	CD14^+^ CD33^+^ HLA-DR^− /low^	Treg cell	MDSCs expansion was closely associated with disease progression (tumor stage, LDH levels and B syndromes).	2022 [51]
	↑PMN-MDSCs	CD10^−^HLA-DR^− /low^			
HL, NHL	↑PMN-MDSCs	CD66b^+^CD33^dim^HLA-DR^−^	/	Higher frequencies of PMN-MDSCs correlated significantly with unfavorable prognostic index scores and a shorter freedom from disease progression.	2016 [60]
AML	↑M-MDSCs	CD14^+^HLA-DR^low/−^ M-MDSC	/	Elevated circulating M-MDSCs in patients with AML were significantly associated with low complete remission (CR) rate, high relapse/refractory rate, and poor long-term survival.	2020 [172]
AML	↑MDSCs	CD11b^+^HLA-DR^−^CD33^+^Lin^−^	↑IL-10; ↓IFN-γ; MUC1 signaling	MDSCs are expanded in patients with AML and contribute to tumor-related immune suppression.	2017 [91]
AML	↑MDSCs	CD11b^+^ CD33^+^ HLA-DR^−^	↑VISTA	VISTA is highly expressed on MDSCs and knockdown of VISTA significantly diminished the MDSCs-mediated inhibition of T cell proliferation.	2018 [93]
AML	↑MDSC-like blast	CD11b^+^CD33^+^HLA-DR^−^	↑iNOS, ARG1	Patients with high MDSC-like blasts at diagnosis showed substantially shorter overall survival and leukemia-free survival relative to low MDSC-like blasts patients.	2020 [173]
AML	↑M-MDSCs	CD14^+^CD33^+^IDO^+^HLA-DR^low^	↑IDO	AMG 330 may achieve anti-leukemic efficacy not only through T-cell-mediated cytotoxicity against AML-blasts but also against CD33 + MDSCs. MDSCs levels could represent a biomarker for the patients’ clinical responsiveness towards an AMG 330-based therapy.	2018 [174]
AML	↑M-MDSCs	CD14^+^HLA-DR^low^	↑IDO, S100A8/9, cEBPβ	Targeting protein palmitoylation in AML could interfere with the leukemogenic potential and block MDSCs accumulation to improve immunity.	2020 [92]
ALL	↑MDSCs	Lin^−^HLA-DR^−^CD33^+^CD11b^+^	/	The correlation between the frequencies of the two immunosuppressive populations, MDSCs and Treg cell in pediatric patients with B-ALL as compared to healthy volunteers.	2018 [175]
B-ALL	↑PMN-MDSCs	CD45^+^CD19^−^HLA-DR^−^CD11b^+^CD33^+^CD15^+^	↑DCs, Direct cell-cell contact, STAT3	PMN-MDSCs levels correlated positively with clinical therapeutic responses and B-ALL disease prognostic markers, including minimal residual disease, and the frequencies of CD20 + and blast cells.	2017 [76]
	↑M-MDSCs	CD45^+^CD19^−^HLA-DR^−^CD11b^+^CD33^+^CD14^+^			
APL	↑M-MDSCs	CD33^+^CD14^+^HLA-DR^−^	↑ARG1, iNOS; IL-13 blocking	Tumour-activated ILC2s secrete IL-13 to induce myeloid-derived suppressor cells and support tumour growth.	2017 [137]
CML	↑PMN-MDSCs	CD11b^+^CD33^+^CD14^−^HLADR^−^	↑ARG1	PMN-MDSCs and M-MDSCs were significantly higher at diagnosis compared to HD and decreased to normal levels after IM therapy. T-reg resulted significantly increased in respect to HD and they directly correlated with PMN-MDSCs.	2014 [88]
CML	↑MDSCs	CD11b^+^CD14^−^CD33^+^	↑ARG1	T cells in CML patients may be under the control of different immune escape mechanisms (MDSCs, Arg1, PD-L1/PD-1 and sCD25) that could hamper the use of immunotherapy in these patients.	2013 [89]
CML	↑PMN-MDSCs	CD11b^+^CD33^+^CD15^+^CD14^−^HLA-DR^−^	↑ARG1, TNFα, IL1β, COX2, IL6	G-MDSCs isolated from CML patients were not able to inhibit T lymphocyte proliferation. MDSCs differentiated in presence of transformed MSC, exhibited an enhanced inhibitory effect on T cell proliferation.	2016 [176]
CLL	↑M-MDSCs	CD14^+^CD11b^+^CD15^−^HLA-DR^−/low^	↑IL-10, TGF-β1	The level of IL-10 and TGF-b1 expression in circulating M-MDSCs in correlation with clinical and laboratory parameters characterizing disease activity and patients’ immune status (Rai stages, ZAP-70-positive, CD38-positive, genetic aberrations).	2020 [83]
CLL	↑M-MDSCs	CD14^+^CD11b^+^CD15^−^HLA-DR^−/low^	↑IDO, IL-10, TGF-Β1	CLL patients with M-MDSCs percentages above 9.35% (according to the receiver operating characteristic (ROC) analysis) had a shorter time-to-treatment and shorter survival time than the group with a lower percentage of M-MDSCs. The M-MDSCs percentage was higher in patients with adverse prognostic factors (i.e., 17p and 11q deletion and CD38 and ZAP-70 expression).	2020 [177]
CLL	↑PMN-MDSCs	HLA-DR^low^CD11b^+^CD33^+^CD15^+^	↑CD124, CD80, PD-L1/2	The balance between the number of PMN-MDSCs and M-MDSCs affects the function of CLL course (high-risk cytogenetics (11q − and 17p−), ZAP70 levels).	2021 [84]
	↑M-MDSCs	HLA-DR^low^CD11b^+^CD33^+^CD14^+^	↑TGFβ membrane protein		
MM	↑PMN-MDSCs	CD11b^+^CD13^+^CD16^+^	↑Inflammatory cytokines	A set of optimal markers (CD11b + CD13 + CD16+) was found, which can accurately detect PMN-MDSCs in MM.	2020 [103]
MM	↑PMN-MDSCs	CD11b^+^CD15^+^CD14^−^HLADR^−^	↑ARG1, TNFα, PROK2	Mesenchymal stem cells support MM cell growth and survival by promoting MDSCs activation.	2016 [97]
MM	↑PMN-MDSCs	HLA-DR^−/low^CD33^+^CD11b^+^CD15^+^CD14^−^	↑CSCs core genes, piRNA-823	There was a correlation between the frequency of PMN-MDSCs and overall survival rate of MM patients.	2019 [101]
MDS	↑MDSCs	CD33^+^Lin^−^HLA-DR^−^	↑TIM3, CEACAM1, IL-1β, IL-18	Suppressed immune function of CD8 + T cells after co-culture of either MDSC or rhCEACAM1 with CD8 + T cells.	2022 [112]
MDS	↑PMN-MDSCs	Lin^−^CD11b^+^CD33^+^CD15^+^	↑CXCR4, CX3CR1	The expansion of MDSCs in MDS correlates with increased risk of disease progression toward AML and also positively correlates with Treg numbers in high risk MDS.	2015 [108]
MDS	↑MDSCs	Lin^−^HLA-DR^−^CD33^+^	↑IL-10, TGF-β	MDSCs in higher-risk MDS have a stronger immunosuppressive effect and might be related to poor prognosis.	2020 [109]
MDS	↑MDSCs	Lin-HLA-DR-CD33+	↑STAT3, ARG1	MDSCs, which are more pervasive in MDS especially in the high-risk patients, can be STAT3-overactivated and facilitate immune escape and disease progression.	2021 [110]
MDS	↑MDSCs	PD-L1^+^CD33^+^CD14^+^	↑PD-L1	Abnormal expansion and activation of MDSC lead to ineffective hematopoiesis.	2019 [113]
allo-HSCT	↑e-MDSCs	HLA-DR^−/low^CD33^+^CD16^−^	↑TGF-β	e-MDSCs prevented acute GVHD in a humanized mouse model in vivo.	2019 [178]
haplo-HSCT	↑PMN-MDSCs	CD45^+^Lin^−^HLA-DR^−/low^CD33^+^CD11b^+^CD14^−^CD66b^+^	↑Soluble factors, IDO, PEG2; Exosomes	PMN-MDSCss contained in the graft exerts an early inhibitory effect on NK cell-mediated GVL activity.	2020 [179]
allo-HSCT	↑M-MDSCs	CD14^+^HLA-DR^−/low^CD80^+^CD86^−^CD40^−^CD64^−^CD16^−^CD163^+^	↑IL-6, TNF‐α	Accumulation of MDSCs in the graft and in peripheral blood result in the successful control of severe aGVHD and long-term survival without influence on risk of recurrence after allo‐HSCT.	2016 [180]

**Fig. 2:**
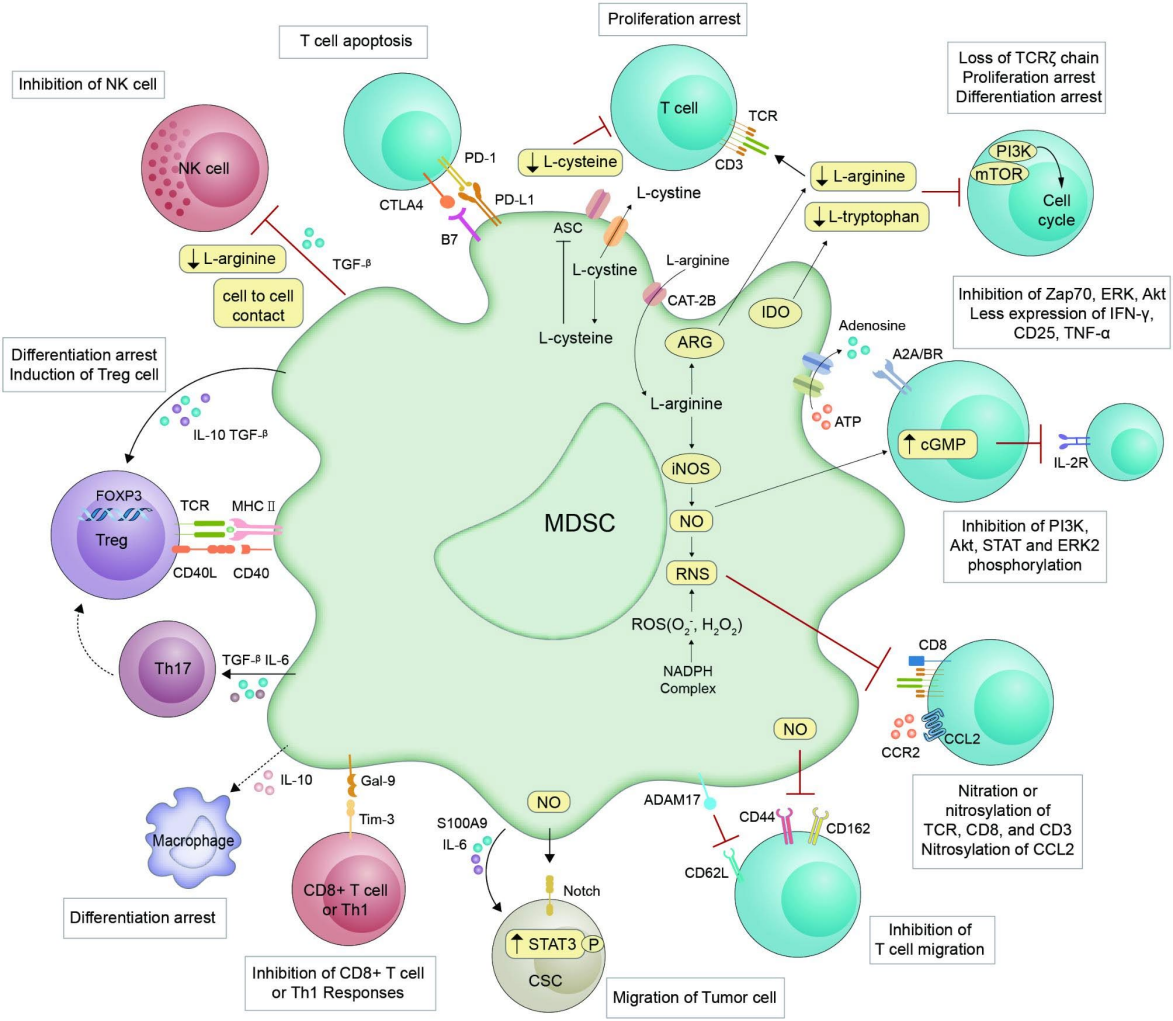
**Immunosuppressive mechanisms and targets of MDSCs.** MDSCs inhibit T-cell activity through distinct mechanisms, including loss of the TCR ζ-chain; nitration of the TCR complex; depletion of amino acids necessary for the T-cell response; the production of adenosine, high levels of NO, RNS, Arg1, and chemokines; the presence of immune checkpoint blockade; and impairment of T-cell homing. Moreover, MDSCs promote Treg and macrophage differentiation and increase FOXP3 expression. Additionally, MDSCs suppress NK cells and CD8^+^ T cells. Finally, tumor-infiltrating MDSCs promote the migration of tumor cells by interacting with cancer stem cells (CSCs).

A core problem in the field of MDSC research is how their amplification, accumulation, and activation proceed. Condamine et al. initially developed the hypothesis of a “two phases model”, which classifies the various cytokines and signaling pathways that have been discovered to be involved in the development and activation of MDSCs into two distinct functional types [39]. The first group of signals mediates the MDSC amplification process and loss of their developmental potential. The second set of signals mediates the activation of MDSCs and acquisition of immunosuppressive function. The first group of signals is mainly propelled by various growth factors produced by tumor-derived and BM-derived growth factors in response to chronic stimulation. These include stem cell factor (SCF), granulocyte colony–stimulating factor (G-CSF), granulocyte-macrophage colony–stimulating factor (GM-CSF), macrophage colony–stimulating factor (M-CSF), and vascular endothelial growth factor (VEGF) [40–43]. This process is also mediated by signal transducer and activator of transcription 3 (STAT3), interferon regulatory factor 8 (IRF8), transcription factor CCAAT enhancer binding protein β (C/EBP β), Rb1, NOTCH, adenosine receptor A2B, and NOD-like receptor family protein 3 (NLRP3) [44]. The second group of signals is proinflammatory cytokines mainly produced by tumor stromal cells, such as IFN-γ, IL-4, IL-6, IL-1β, and CXCL1 [45], which are responsible for inducing the suppressive activity of MDSCs via NF-κB, PI3K-AKT, STAT1, and STAT6 [7, 45]. These signals can induce MDSCs to express inhibitory molecules such ARG-1, iNOS, NOX-2, COX-2, TGF-β, and IL-10 [7]. MDSCs need to undergo myelopoiesis in the BM and lymphatic organs, mobilize to the periphery, and then develop and exercise their immune properties in the TME. The two-phase model is centered on the biological activity of MDSCs. Nevertheless, a recent article highlights the migratory properties of MDSCs, proposing an increase in MDSC homing to form four-step events (steps I-IV) on a two-step basis [12]. Chemokines can mediate the migration of immune cells into the TME [46]. The immunological maintenance functions of MDSCs are coordinated by a variety of crucial chemokines, including CCR2 and CCR5 [47, 48]. Additionally, the recruitment and activation of MDSCs is also regulated by miRNAs and exosomes in the TME [49]. The MDSC amplification and activation of these models overlap, which is exceedingly complicated and involves a number of variables [7]. The immunosuppressive mechanisms of MDSCs are shown in Fig. 2.

## The function of MDSCs in hematologic malignancies

The transition of the TME to an inhibitory area is facilitated by MDSCs. Understanding how MDSCs are expressed in diverse hematological malignancies and their role in clinical prognosis can help inspire new concepts and ideas for future targeted treatments.

### Lymphoma

Lymphoma is a malignant tumor originating from lymphoid tissues and lymph nodes and is characterized by abnormal proliferation of B cells and T cells. It is mainly divided into Hodgkin and non-Hodgkin lymphomas [50]. The properties of lymphomas and solid tumors are more similar to those of other hematological malignancies. Several studies have proven the clinical correlation between MDSCs and most lymphoma subtypes [51–57]. According to a data analysis by Wang et al., patients with newly diagnosed and relapsed diffuse large B-cell lymphoma (DLBCL) had considerably higher levels of M-MDSCs in their peripheral blood, and the levels of M-MDSCs in newly diagnosed DLBCL patients were negatively correlated with overall survival (OS), positively correlated with tumor progression, and correlated with the International Prognostic Index (IPI) score [58]. It was discovered that only M-MDSCs (CD14^+^HLA-DR^low^) are associated with event-free survival and regulatory T-cell (Treg) number in DLBCL in the study of Imheane Azzaoui et al. [55]. Patients with relapsed/refractory (R/R) DLBCL had higher concentrations of MDSCs and Tregs than the healthy group, and reduced levels of Tregs and MDSCs correlated with a better OS [59]. M-MDSCs are more numerous in DLBCL, and G-MDSCs are even considered nonfunctional [58]. It was discovered that IL-35 could lead to the accumulation of CD11b^+^Gr1^+^ myeloid cells and that using a neutralizing antibody to remove IL-35 directly from mouse experiments could help lessen the buildup of M-MDSCs in mice with Ly8 DLBCL tumors [58]. However, the precise mechanism is still unknown. Since the majority of blood biomarkers in DLBCL are linked to the tumor microenvironment, specifically to myeloid cell biology, further identification of the MDSC subgroups is crucial [55]. However, Marini et al. reported that the frequency of CD66b^+^CD33^dim^HLA-DR^−^ PMN- MDSCs in 124 patients with B-cell lymphomas (including CHL and NHL) was higher than that in the healthy control group, and the depletion of CD66b^+^ cells in PBMCs of patients restored the proliferation of autologous T cells in vitro [60]. More research is necessary to determine why M-MDSCs are prominent only in DLBCL.

The expansion of MDSCs is closely related to the disease progression of lymphoma and has an indicative role in the prognosis of lymphoma patients [51, 58]. Immature CD34^+^ MDSCs are considered a new potential prognostic marker in CHL patients. In PB samples from 60 newly diagnosed HL patients, all three MDSC subtypes, including CD34^+^, were increased. The MDSC counts were lower in patients who achieved a complete response (CR) after chemotherapy than in patients who did not respond [53]. Additionally, studies have discovered that elevated PMN-MDSCs (CD11b^+^CD15^+^CD33^int^) in the duodenum dampen the development of enteropathy-associated T-cell lymphoma (EATL) by promoting antitumor T-cell immunity [61]. According to a study of 32 patients with the disease, the percentage of circulating HLA-DR^−^/CD33^+^/CD11b^+^ MDSCs was higher in extranodal NK/T-cell lymphoma (ENKL) patients than in healthy controls, and they were an independent predictor of disease-free survival (DFS) and overall survival (OS) [62]. In addition, ENKL-MDSCs exhibited high levels of ARG1, iNOS, and IL-17; moderate levels of TGF-β and IL-10; and low levels of CD66b. These characteristics greatly inhibited the proliferation of anti-CD3-induced CD4^+^ T cells but only weakly inhibited the proliferation of CD8^+^ T cells.

In a study of mice with RMA-S lymphoma, Shlecker et al. demonstrated that populations of PMN-MDSCs and M-MDSCs were present in high numbers in the peripheral blood, spleen, and tumor tissues of tumor-bearing mice [63]. Research on tumor-bearing adiponectin knockout (APNKO) mice shows that adiponectin is an important regulator of EL4 lymphoma-carrying mouse MDSC amplification, in which G-CSF plays an important role in MDSC differentiation [64]. MicroRNA (miR)-30a increased microRNA expression in both PMN-MDSCs and M-MDSCs in B-cell lymphoma model mice [65]. After transfection with miR-30a mimics, the differentiation and capacities of MDSCs were significantly increased via upregulation of ARG-1. Decreased SOCS3 expression and activation of Janus kinase-signal transducer and activator of transcription 3 signaling promote MDSC differentiation and activity. The factors IL-13, IL-10, and S100A12 and increased PD-L1 expression are all implicated in the suppression of bone marrow-dependent T cells in DLBCL [59]. However, ARG1 and IDO1 associated with MDSC amplification do not play a regulatory role in DLBCL [58]. This lack of regulatory inhibition has not been explained and verified. Among solid tumors [66] or in subcutaneous lymphoma models [67], MDSCs can exert their inhibitory effect on tumor-specific T-cell responses mediated by TGF-β [68], arginine [69], and/or nitric oxide [70]. Using the A20 B-cell lymphoma model to negate the dominant role of TGF-β on Treg amplification, MDSCs can inhibit the effect of T-cell function by MDSCs through arginine metabolism [71]. However, sildenafil treatment resulted in decreased IL4R levels, reduced Treg amplification, and restored tumor-inducing T-cell function.

MDSCs and effector Th17 cells are involved in immune dysregulation during the development of gastric mucosa-associated lymphoid tissue (MALT) lymphoma and are accompanied by increased expression of ARG1, iNOS, IL-23, IL-1β, and the chemokine CCL20/CCR6 [72]. However, in non-Hodgkin lymphoma (NHL), MDSCs act on NK cells through the immunosuppressive effect of IL-10 but not on T cells. Moreover, CD27^+^CD11b^+^ NK cells negatively regulate the expansion of Gr1^+^CD11b^+^Ly6G^med^Ly6C^med^ MDSCs and MDSC expression of MHC class II, CD80, CD124 and CCR2 in the EL4 mouse lymphoma model [73]. In addition to PD-L1, other immune checkpoints, such as TIGIT and c-Rel, have also been verified to have tumor-promoting effects on MDSCs in lymphoma, which may become an immunotherapeutic strategy targeting MDSCs [74, 75]. It can be hypothesized that MDSCs have important clinical significance for lymphoma patients. However, due to the high heterogeneity of lymphomas and MDSCs, the pathological mechanism and role of target MDSCs in lymphoma have become complex, and more in-depth research is still needed.

### Leukemia

Leukemia is a malignant clonal disease of hematopoietic stem and progenitor cells. Liu et al. confirmed that the PMN-MDSC population in the peripheral blood and BM of children with B-ALL was significantly increased, and the level of PMN-MDSCs was positively correlated with the therapeutic response and the prognosis markers of B-ALL disease, including minimal residual disease and the frequency of CD20 + and primitive cells [76]. During induction treatment, patients with B-ALL showed higher MDSC and Treg cell levels than those with an early diagnosis of the condition [76]. The levels of MDSCs and Tregs in different stages of leukemia vary, and some researchers have proposed that MDSCs and Tregs are independent predictors of B-ALL progression [77]. Using Affymetrix microarray technology, Labib’s group found that miRNAs specifically induced the recruitment of MDSCs and Tregs, and these miRNAs could be potential biomarkers as they affected the progression of B-ALL [78]. This view was also verified in DLBCL [79]. Recently, Grazioli et al. demonstrated MDSC amplification through the Notch pathway by establishing a T-ALL transgenic mouse model of Notch3 [80].

In a study including 49 CLL patients, Liu et al. found that all CLL patients had markedly increased CD14^+^HLA-DR^low/−^ MDSCs, which lessen the CD4^+^ T-cell immunological response and promote CLL cell development [81]. In addition, the level of M-MDSCs in CLL was basically related to the frequency of the deletion of the prognostic markers CD38, ZAP70, 11q22.3 and/or 17p13.1 [82, 83]. The ratio of PMN-MDSCs to M-MDSCs has an impact on CLL development. The given explanation is that M-MDSCs mediate the immune suppression function of T cells by TNF-α [84]. Through the construction of the CLL E-TCL1 mouse model, it was found that IDO1 and secretory IgM promote the expansion of MDSCs, but the inhibition of IDO1 could not reduce the recurrence of leukemia [85, 86]. Patients with AML and CML are reported to have elevated numbers of MDSCs in the bone marrow, and these levels diminished after treatment [87, 88]. The progression of tumors relies on elevated MDSC levels in Sokal high-risk patients, along with elevated soluble CD25, arginase 1, and PD-L1 expression [89]. The levels of MDSCs in the high minimal residual disease (MRD) group were significantly higher than those in the middle/low MRD groups in 123 AML patients [87]. The expression of MDSCs also plays a prognostic role in myeloid leukemia.

Extracellular vesicles (EV) are important vectors for MDSC amplification in AML and CML [90, 91]. It was proven that MUC1 oncoprotein-driven c-MYC expression in EVs in AML resulted in downstream MDSC proliferation using the TIB-49 AML-transplanted C57BL/6 mouse model [91]. Additionally, the CD14^+^HLA-DR^low^ phenotype and functional modification of monocytes produced by AML-EVs are significantly influenced by the Akt/mTOR pathway. The activation of Toll-like receptor 2 by palmitoyl protein on the surface of AML-EVs supports EVs as the initial event in the AKT/mTOR-dependent induction of MDSCs [92]. Another negative regulator of cancer immune evasion is V-domain Ig inhibitor of T-cell activation (VISTA). According to Wang’s research, MDSCs were shown to be increased in the peripheral blood of AML patients, and VISTA knockdown significantly decreased the ability of these cells to inhibit PD-1-expressing CD8^+^ T cells in AML [93]. Recently, it was found that the expression of GPX1 in AML was positively correlated with MDSC, monocyte and T-cell depletion scores, and interestingly, it was also associated with immunosuppression checkpoints (TIM3/GAL-9, SIRPα, and VISTA), and these checkpoints participate in the immunosuppressive effects of MDSCs [94]. These potential pathways and targets of mechanistic research provide theoretical support for immune drug application.

### Multiple myeloma

Multiple myeloma (MM) is a clone of malignant plasma cells in the bone marrow with extramedullary infiltration. Similar to other tumors, the number of MDSCs is increased in MM [95]. This is accompanied by higher ARG-1, iNOS, ROS, TNF-α, and IL-10 levels [96–98]. According to previous studies, M-MDSC levels have a negative correlation with therapeutic response and a positive correlation with MM recurrence [95]. PMN-MDSCs are found in higher numbers in the BM and PB of MM patients, and research has related them to the progression and treatment resistance of MM [99, 100]. In MM cells, G-MDSCs enhanced the side population, sphere formation, and expression of cancer stem cells (CSCs) core genes [101]. Furthermore, G-MDSCs induce piRNA-823 expression, promote DNA methylation and heighten tumorigenic potential [101]. The secretion of C-C motif chemokine ligand 5 (CCL5) and macrophage migration inhibitory factor (MIF) by myeloma cells is a prerequisite for inducing MDSCs in MM [102]. Recent studies have suggested that the (CD11b/CD13/CD16) PMN-MDSC phenotype can be used to accurately monitor MM and its clinical transformation [103]. In addition to promoting the growth of MM cells by secreting inhibitory factors, MDSCs can also partly promote tumor growth through AMPK activation [104]. In addition, mesenchymal stem cells promote MDSCs by inhibiting T-cell proliferation and IFN-γ production to enhance the immunosuppressive effect of MDSCs [105]. MDSC subtypes have different functions in MM. PMN-MDSCs can secrete angiogenesis-related factors to promote angiogenesis, while M-MDSCs can be osteoclast precursors and participate in the pathological mechanism of osteolytic bone destruction [106].

### Myelodysplastic syndrome

Myelodysplastic syndrome (MDS) is a heterogeneous clonal disease of hematopoietic myeloid directed stem cells or pluripotent stem cells. It is characterized by ineffective hematopoiesis, morbid hematopoiesis, and conversion to AML. Elevated MDSC levels play a central role in the pathogenesis of MDS and dysregulation of immune surveillance and are connected with the risk of MDS progression to AML and a poor prognosis [107, 108]. It has been demonstrated that in MDS patients, the population of MDSCs in peripheral blood is significantly lower in very low/low-risk patients than in medium/high/very high-risk patients [108]. In both high-risk and non-low-risk patients, there is a positive relationship between the proportion of Tregs and MDSCs. The data further showed that tumor-derived MDSCs expressed CXCR4 and CX3CR1, which facilitate the migration of MDSCs to the bone marrow. It is worth noting that M-MDSCs in peripheral blood more often express CX3CR1 and CXCR4 at higher levels than PMN-MDSCs. Further studies showed that the ratios of IL-10/IL-12 and TGF-β/TNF-α in high-risk MDS patients were significantly higher than those in low-risk MDS patients and normal controls [109]. The ratio of TGF-β/TNF-α in MDSCs was positively correlated with the percentage of primitive cells and negatively correlated with the percentage of CD3^+^CD8^+^ T-lymphocytes. MDSCs in high-risk MDS have a stronger immunosuppressive effect, which may be related to a poor prognosis.

The equilibrium of MDSC immunity in MDS was examined utilizing the ratios of IL-10/IL-12 and TGF-β/TNF-α. In high-risk MDS patients, MDSCs show high levels of active STAT3 and CCR2, and the STAT3-ARG1 pathway may be the significant signaling pathway regulating MDSC-mediated CD8^+^ T-cell immunosuppression [110]. In addition to the immune checkpoint protein TIM3 ligand Gal-9 [111], another TIM3 ligand, CEACAM1MDS, is also increased in the MDSC-mediated CD8^+^ T-cell failure pathway in MDS [112]. Compared with healthy donors, the expression of PD-1 on HSPCs and PD-L1 on MDSCs is higher in MDS, and this checkpoint was also found to be activated in S100A9 transgenic (S100A9Tg) mice [113]. The PD-1/PD-L1-related effects affect the hematopoietic pathways of MDS, and blocking PD-1 or PD-L1 helps reverse the ineffective hematopoietic environment triggered by MDSCs. The accumulation of CD33^+^HLA-DR^−^Lin^−^ MDSCs has important significance in the pathogenesis of MDS. Eksioglu et al. reported that the interaction of CD33-S100A9 triggers the inflammatory signaling cascade reaction leading to ROS release, which is related to DNA damage [113]. In MDS patients, an anti-CD33 monoclonal antibody can reduce MDSC levels, thus blocking the downstream signal transduction of CD33 and preventing the secretion of immunosuppressive cytokines, thereby reducing ROS levels and DNA damage.

### Myeloproliferative neoplasms

CML, a myeloproliferative tumor, is characterized by the employment of the aberrant oncoprotein BCR-ABL. Giallongo’s group reported that CML cells encourage MDSC multiplication by releasing soluble substances and exosomes, producing an immune-tolerant milieu that causes T-cell anergy and encourages tumor growth [114]. CML is described in the leukemia section above and will not be repeated here.

The majority of cases of myeloproliferative neoplasms (MPNs), which are clonal myeloid malignancies produced from stem cells, result from three mutually exclusive mutations (JAK2V617F, MPL, and CALR), which have a distinct somatic mutational profile [115]. Recent studies have shown that MDSCs and PD-L1 are involved in the immune escape mechanism of proliferative tumors [116, 117]. In contrast to Wang’s work, which showed increased PD-1 and PD-L1 expression in MPN, Kundra’s study found no increase in the expression of these proteins in MPN. According to additional research, MPN was categorized into groups by Wang et al. The levels of PD-1 and PD-L1 in CD4^+^CD8^+^CD14^+^CD34^+^ progenitor cells when PV, ET, and MF were separated into MPN were significantly different from those of the control group. In the study of Kundra et al., MPN was not grouped, and the selected cells were CD4^+^CD8^+^CD14^+^ MNC cells. In both studies, MDSCs increased and exerted immunosuppressive functions. Given that MPN represents a stem cell disease, it is envisioned that anti-MDSC agents in combination with ICIs such as PD-1 and PD-L1 in PH (-) MPN may be necessary.

## The implications of MDSCs in the treatment of hematologic malignancies

**Fig. 3:**
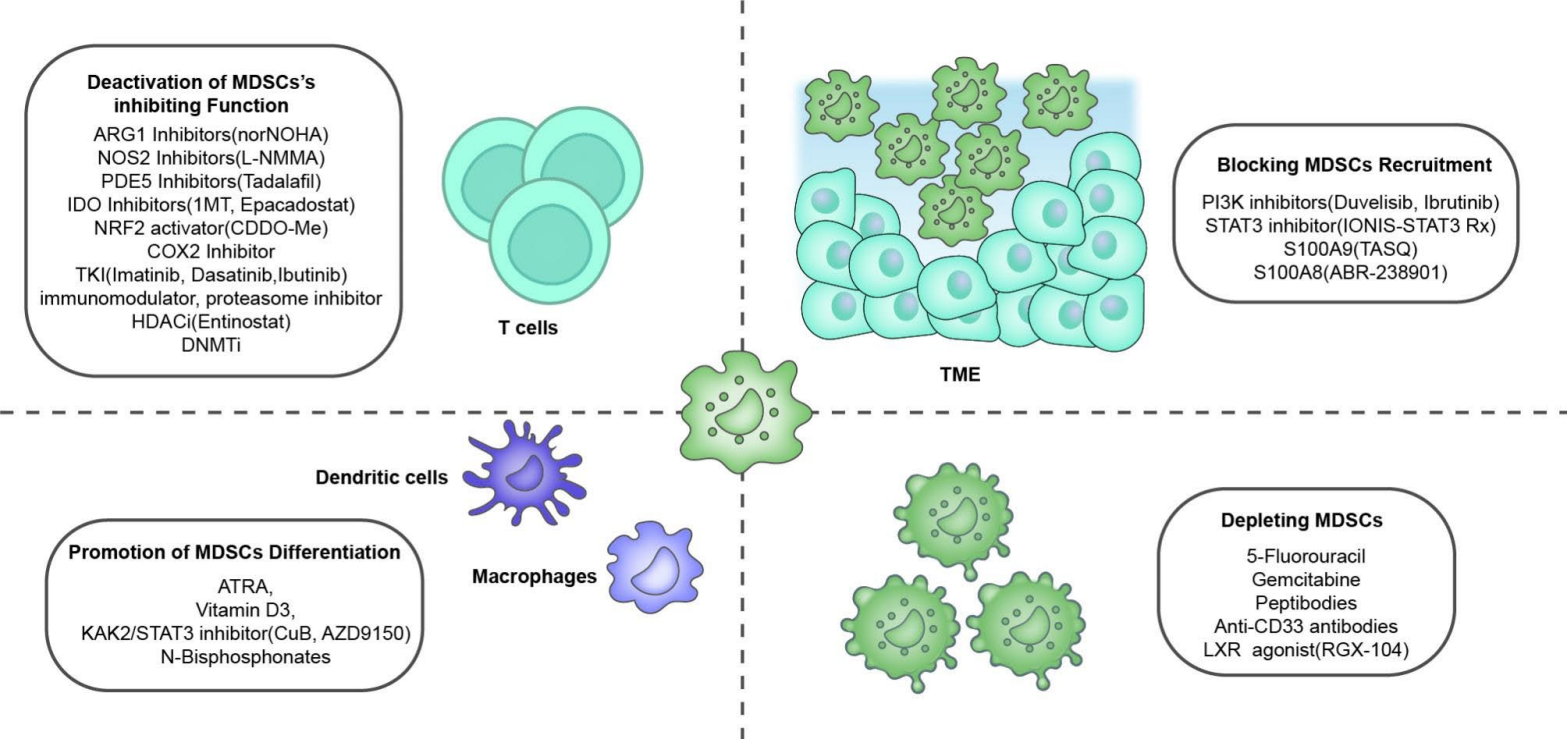
**Therapeutic agents against MDSCs classified by mechanism in hematological malignancy.** The main approaches to target MDSCs include [1] depleting MDSCs; [2] blocking MDSC recruitment to the tumor microenvironment (TME); [3] promoting the differentiation of MDSCs into mature myeloid cells; [4] blocking MDSC-mediated immunosuppression

Hematological tumor therapy mainly includes chemotherapy, targeted therapy, and immunotherapy. As early as the beginning of the 21st century, studies have shown that all-trans retinoic acid (ATRA) can induce the differentiation of immature myeloid cells in tumor patients and reduce their immunosuppressive function [118]. A growing number of potential drugs have been studied for targeting MDSCs [119], most of which are more effective in tumor control than existing treatment regimens Fig. 3. The treatments aimed at MDSCs are mainly divided into four categories: reduction of the number of MDSCs, inhibition of MDSC differentiation, inhibition of MDSC recruitment, and MDSC inactivation (Table 2). A valuable research direction for MDSC-related cell therapy is the prevention and treatment of graft-versus-host disease after hemopoietic stem cell transplantation (HSCT).

**Table 2:** Summary of clinical trials targeting MDSCs in hematological malignancy

NCT number	Drug name	Target	Indications	Last reported status	Phase
NCT01393106	Idelalisib	PI3K	Hodgkin lymphoma	Completed	II
NCT01374217	Tadalafil, Lenalidomide, Dexamethasone	PDE5	MM	Recruiting	II
NCT02916979	Fludarabine, Busulfan	/	Leukemia, MDS	Active, not recruiting	I
NCT03480360	Cyclophosphamide, Fludarabine	/	Leukemia	Recruiting	III
NCT01563302	IONIS-STAT3Rx	STAT3	DLBCL lymphoma	Completed	I/II
NCT03214666	GTB-3550 TriKE™	CD33	Leukemia	Recruiting	I/II
NCT02076451	DS-8273a	TRAIL-R2	Lymphoma	Completed	I
NCT04405167	Tasquinimod, IRd chemotherapy	S100A9	MM	Recruiting	I
NCT04915248	Daratumumab, Bortezomib, Dexamethasone	CD38	Plasmablastic Lymphoma	Recruiting	II
NCT02846376	Nivolumab, Ipilimumab	CTLA-4, PD-1	AML, MDS	Active, not recruiting	I
NCT04147533	Dasatinib, Nilotinib	BCR-ABL	CML	Recruiting	II
NCT05032820	Lenalidomide, bb2121	/	MM	Recruiting	II
NCT01675141	Lenalidomide	/	MM	Completed	II
NCT05293912	SG2501	CD38	Hematological Malignancy	Recruiting	I
NCT02936752	Entinostat	HDAC	MDS	Active, not recruiting	I
NCT02922764	RGX-104, Nivolumab, Ipilimumab	LXR	advanced solid tumors, lymphoma	Recruiting	I
NCT01347996	histamine dihydrochloride, IL-2	NOX2	AML	Completed	IV
NCT03144245	AMV564	CD33	AML	Completed	I
NCT03516591	AMV564	CD33	MDS	Completed	I

### Depleting MDSCs

MDSCs are cells that have a short survival time in the blood. Conventional cytotoxic chemotherapy, such as 5-fluorouracil, gemcitabine, tyrosine kinase inhibitors (TKIs), or targeting S100A via a therapeutic peptide Fc fusion protein, has been shown to deplete MDSCs in solid tumors and to promote antitumor effects [120]. 5-Fluorouracil and gemcitabine selectively induce the apoptosis of MDSCs in mouse models and initiate antitumor effects [121–123]. Additionally, gemcitabine combined with DC-mediated immunotherapy markedly enhances the therapeutic effect against lymphoma, indicating the potential of combination therapy for treating this malignancy [122]. Sasso et al. showed that a low-dose encapsulated gemcitabine formulation can selectively target M-MDSC subsets and reduce tumor-associated immunosuppression in the TME using the E.G7-OVA lymphoma model [124]. After TKI treatment of CML patients, the proportion of PMN-MDSCs and serum ARG1 and iNOS levels decreased significantly, with the proportion of effector NK cells increasing [125]. However, the treatment of CML with the TKI dasatinib resulted in a significant decrease in M-MDSCs [126]. Peptide-Fc fusion (peptide bodies) can specifically reduce the number of MDSCs in the blood, spleen, and tumor to reduce immune suppression without affecting the type of proinflammatory immune cells, which was also verified in different mouse lymphoma models (A20, EG7, and EL4) [127].

A more elaborate approach uses the antibody CD33. The humanized Fc-engineered CD33 monoclonal antibody (BI 836,858) reduces MDSCs through antibody-dependent cytotoxicity and blocks the secretion of immunosuppressive cytokines [128]. Reactive oxygen species (ROS) can lead to genomic instability when the CD33 signaling pathway is activated, whereas BI 836,858 decreases the levels of double strand breaks and adducts in addition to ROS [128]. Further research found that treatment with this monoclonal antibody mainly removes the MDSC wall in the tumor environment, leaving beneficial immune cells relatively unaffected, which has shown satisfactory results in subsequent clinical trials [129]. CD33 is specifically overexpressed in AML cells but not in hematopoietic stem cells or mature granulocytes, making CD33 a potential therapeutic target. Following the dose adjustment of gemtuzumab ozogamicin (GO), a humanized anti-CD33 monoclonal antibody, there was renewed interest in CD33 targeted therapy [128]. The drug AMV564 is a CD33/CD3 dual-specific T-cell activator. At present, clinical studies are being conducted on patients with advanced solid AML, MDS, or malignant tumors (NCT03144245, NCT03516591, and NCT04128423) [130]. At a tolerable dose, AMV564 leads to a decrease in MDSCs, an increase in cytotoxic cell activation, and a decrease in tumor burden. When AMV564 is used as a single drug or in combination with immunotherapy targeting checkpoints, continuing clinical evaluation of the drugs is encouraged due to the reduction in CD33^high^ MDSCs and improvement in cytotoxic T-cell activation in malignant tumors [130]. In addition to CD33-targeted biotherapeutics (CD3^+^CD33^+^ or CD16^+^CD33^+^) [130, 131], CD123-targeted antibodies and BiABs have high binding affinity for targets on MDS clones and MDSCs, which also shows their clinical potential for treating high-risk MDS patients [132].

Liver X receptors (LXR and isoforms) modulate cholesterol homeostasis, which has an impact on the survival and proliferation of leukemia cells [133]. The LXRβ agonist RGX-104 abolishes tumors by reducing the number of bone marrow-derived suppressor cells and increasing the number of antigen-specific T cells [134]. Preliminary results from an ongoing phase I clinical trial (NTC02922764) show that RGX-104 causes a decrease in MDSCs in patients with metastatic solid cancer or lymphoma, accompanied by cytotoxic T lymphocyte (CTL) activation.

### Promotion of MDSC differentiation

Vitamin A and vitamin D3 trigger the differentiation of MDSCs [135, 136]. The first acute promyelocytic leukemia (APL) medication, all-trans retinoic acid (ATRA), eliminates PMN-MDSCs while stimulating MDSC development into macrophages and dendritic cells. In patients with APL, tumor-activated group 2 innate lymphoid cells (ILC2s) secrete IL-13 to induce M-MDSCs (CD33^+^CD14^+^HLA-DR^−^) and support tumor growth, while ATRA treatment reverses the increase in ILC2-MDSCs in APL [137]. Recently, lipid nanoformulations have been used as carriers of ATRA, improving the bioavailability of ATRA and the tumor treatment efficiency [138]. A selective inhibitor of JAK2/STAT3 signaling, cucurbitacin B (CuB), promotes the differentiation of MDSCs into DCs [139]. Although the STAT3 antisense oligonucleotide AZD9150 causes a decrease in MDSCs and an increase in DCs, whether this targeting drug causes MDSC differentiation needs to be verified experimentally [140]. JAK2/STAT3 signaling is a potential pathway for MDSC-targeted therapy. Adjuvant epigenetic therapy for solid tumors not only inhibits the movement of MDSCs into tissue but also destroys the premetastatic niche by promoting the differentiation of MDSCs into a more interstitial macrophage-like phenotype [141]. However, the mechanism of differentiation in hematological tumors needs to be verified. In addition, nitrogen-containing bisphosphonates (N-bisphosphonates), such as zoledronic acid, have the ability to promote the differentiation of MDSCs into mature cells in murine models of solid tumors, independent of their ability to inhibit phenylpropylation [142]. N-Bisphosphonates play a major role in the supportive treatment of MM patients, but the effect of this drug on MDSCs in MM remains to be explored.

### Blocking MDSC recruitment

PI3K inhibitors have recently been shown to reduce tumor and spleen MDSCs in a mouse model [143]. The inhibitors duvelisib and ibrutinib are in phase II clinical trials for the treatment of lymphocytic leukemia (NCT04209621), and the PI3K inhibitor idelalisib is being tested in a Hodgkin’s lymphoma phase II clinical trial (NCT01393106). In several cancer types, STAT3 is a key transcription factor for MDSC amplification and upregulates arginase 1, S100A8, and S100A9 [144]. There is an ongoing clinical trial of the STAT3 inhibitor IONIS-STAT3RX for the treatment of DLBCL lymphoma (NCT01563302) [7]. Tasquinimod (TASQ) is an investigational drug targeting MDSCs through the S100A9 protein. A recent phase 1 trial (NCT04405167) to determine the maximum tolerated dose (MTD) and optimal treatment dose of TASQ in MM patients investigated the MTD of TASQ in combination with standard regimens of ixazomib, lenalidomide, and dexamethasone (IRd) in oral myeloma. In a study of the small molecule S100A8 inhibitor ABR-238,901 combined with bortezomib in the treatment of MM, the use of the immune mouse 5T33MM model proved that the treatment did not directly affect the accumulation of MDSCs but decreased the expression of the cytokines IL-6 and IL-10 in MDSCs with a reduction in tumor load [145].

### Blocking MDSC-mediated immunosuppression

MDSCs upregulate the expressions of ROS, iNOS, ARG-1, and other immunosuppressive factors and decrease the antitumor activity of T cells. Therefore, these factors have become important therapeutic targets. Activated MDSCs express significant amounts of ARG-1 and NOS2, and inhibitors of both enzymes (L-NMMA for NOS2 and norNOHA for ARG-1) reversed the MDSC immunosuppression mechanism in MM and lymphoma models [96, 98, 146]. Noonan et al. demonstrated that tadalafil, a phosphodiesterase 5 (PDE5) inhibitor, reduces the expression of ROS, ARG-1, and iNOS in the MDSCs of patients with advanced relapsed/refractory MM and restores the antitumor immune response of T cells after treatment [147]. Targeting indoleamine 2,3-dioxygenase (IDO1) has therapeutic effects not only on MDSCs but also on Tregs [148]. A preclinical treatment study with the Eµ-TCL1 mouse model of CLL treated with two IDO1 inhibitors, namely, 1-methyl-D-tryptophan (1-MT) and epacadostat, found that these two inhibitors had only a slight impact on the progression of CLL. The activity of aromatic hydrocarbon receptor (AHR) and the expression of IL4Il may explain why inhibitors of IDO1 slightly affect the progression of CLL [86]. The synthetic triterpenoid C-28 methyl ester of 2-cyano-3,12-dioxooleana-1,9,-dien-28-oic acid (CDDO-Me) is an NRF2 activator that eliminates the immunosuppression of MDSCs by reducing the levels of ROS and nitrotyrosine in the EL4 mouse tumor model [149–151]. A COX2 inhibitor can reduce the amount of e-MDSCs and their immunosuppressive function in mesothelioma, but this effect still needs to be confirmed in hematological tumors [152]. The TKIs imatinib and dasatinib reduce the levels of MDSCs and ARG-1, MPO, IL-10, and MDSC inhibitory effects in CML and regulate T cells [147, 153]. However, the TKI ibutinib only acts on PMN MDSCs and Th cells in CLL, hindering tumor progression [84]. High concentrations of S100A9, IL-10, and TGF-β produced by MDSCs in MDS BM matrix-β activate the proto-oncogene MYC. This induces PD-L1 in tumors to promote immune escape. Bortezomib [154], lenalidomide + anti-PD-1 [155] or DCs + lenalidomide + anti-PD-1 [156] all cause a decrease in MDSC numbers in MM and an improvement in the tumor microenvironment. However, some studies have concluded that these proteasome inhibitors and immunomodulators cannot deplete MDSC counts, and their clinical efficacy is not attributed to MDSCs [96, 156].

There is still controversy about the actions of these drugs on MDSCs and the immune microenvironment that needs further research. It is crucial to note that in recent years, attention has also been focused on the characteristics of BCMA antigen targeting, CD38 antigen targeting, CD123 antigen targeting, and bispecific antibodies (BsAB) in therapeutic strategies and their effect on the evolution of multiple myeloma (MM) through MDSCs [157]. Epigenetic therapies using low-dose histone deacetylase inhibitors (HDACis) or DNA methyltransferase inhibitors (DNMTis) have been shown to reduce the number of MDSCs in various preclinical models of solid tumors [141]. They disrupt this premetastatic microenvironment and inhibit metastasis and may be an adjunctive approach to cancer treatment [141]. A recent phase 1 study combined the protein deacetylase inhibitor entinostat plus clofarabine to treat low-risk Philadelphia chromosomal negative (newly diagnosed older adults or adults with relapsed and refractory disease) acute lymphoblastic leukemia or biphenotypic leukemia. However, this study lacked a specific investigation of MDSCs [158].

### Transplantation

The clinical importance of allogeneic hematopoietic stem cell transplantation (allo-HSCT), a therapeutic treatment for hematological malignancies, is substantial. The most frequent complication of allo-HSCT is acute and chronic graft versus host disease (aGVHD/cGVHD), which has a high incidence rate and mortality and has a significant detrimental impact on the efficacy of the procedure and the survival of transplant patients. The pathogenic process of GVHD occurs when alloantigens on host antigen-presenting cells (APCs) activate donor T lymphocytes, which then attack recipient tissue via Fas-FasL interactions and TNF-α [159]. As for their inhibitory effects on alloreactive T-cell priming and growth as well as the induction of Tregs, there has been increasing interest in the contribution of donor MDSCs to GVHD management. MDSCs can prevent the development of GVHD in HSCT and preserve the graft vs. leukemia (GVL) effect of grafts. In addition to suppressing alloreactive responses mediated by T lymphocytes and NK cells during graft infusion [160–163], it has been hypothesized that MDSCs will also impact the Th17/Tc17-Treg balance in allo-HSCT grafts and play a role in the etiology of cGVHD [164]. Furthermore, a significant number of mouse models have demonstrated that MDSCs can inhibit GVHD toxicity by strongly inhibiting a T-cell mediated allogeneic reaction and enhancing Treg activity [159]. MDSCs can be used as a new cell-based therapy that is different from their role in other blood tumors. MDSCs derived from the G-CSF mobilization program can strongly inhibit a T-cell mediated allogeneic reaction and enhance Treg activity, thereby inhibiting GVHD toxicity [165]. At the same time, GVL activity was maintained by selectively inducing NKG2D^+^ CD8^+^ memory T cells [166]. In the study of Zhang et al., CD115^+^Gr-1^+^F4/80^+^ MDSCs were identified, and it was found that, compared with the commonly defined Gr-1^+^CD11b^+^ MDSCs, CD115^+^Gr-1^+^F4/80^+^ cells showed stronger inhibition ability and induced the growth of CD4^+^CD25^+^Foxp3^+^ T regulatory cells (Tregs) in tumor-bearing mice. More importantly, CD115^+^ MDSCs induced the proliferation of NKG2D^+^CD8^+^ T cells, while the clearance of allogeneic lymphoma cells required the expression of NKG2D on donor T cells. This information provides a new treatment option for GVHD. Extracting large numbers of MDSCs is challenging because MDSCs are found in low numbers in healthy human tissues [165]. Interestingly, recent studies have found that combined administration including ATRA, paclitaxel, vitamin D, and IL-2, can be able to induce MDSC differentiation by blocking their immunosuppressive activity [167]. By pretreating the recipient with cytokines, murine MDSCs can also be induced. A new MDSC subgroup (MDSC-IL-13) produced by exogenous IL-13 exposure was found. Although both MDSCs and MDSC-IL-13 inhibit the lethality of GVHD, MDSC-IL-13 is more effective. This inhibition is attributed to the upregulation of ARG-1 and PD-L1 [168]. Starting before hemopoietic cell transfer (HCT) of allogeneic mice, the cells were pretreated with exogenous IL-33 to increase MDSCs and Tregs and inhibit GVHD lethality [169]. Treatment with β-galactose lectin-9 (Gal-9), a soluble lectin family member bound to galactoside, can increase MDSCs (G9-MDSCs) and inhibit the proliferation and activation of T cells. Infusion of G9 MDSCs into the graft successfully controls the long-term survival of severe aGVHD in allogeneic bone marrow transplant mouse models [170]. Recent studies have found that peg-G-CSF mobilized leukocyte isolated MDSCs can reduce severe acute GVHD compared with peg-G-CSF mobile grafts after allo-HSCT [171]. The specific mechanism of the MDSC effects on GVHD and GVL has not been fully determined. Moreover, the overwhelming immunological response that drives ongoing GVHD was not under MDSC control. Consequently, more research is required to confirm the efficacy and safety of MDSCs as a targeted therapy for GVHD.

## Conclusion

MDSCs play a pathogenic role in the immunosuppressive tumor microenvironment, which constitutes an obstacle to the efficacy of immunotherapies such as CAR-T and ICIs. However, the complex phenotypic markers and lack of specific recognition of MDSCs, as well as the differences in pathogenesis in different tumors, show that the in-depth research and targeted treatment of MDSCs in hematological tumors still pose challenges. The development and popularization of multiomics technology and single-cell sequencing will help to more deeply understand the evolution of MDSCs and their specific regulatory functions in different blood tumor models. Targeting MDSCs to reshape the immunosuppressive microenvironment may be a promising direction for precise immunotherapy.

## Data Availability

The data used to support the findings of this study are included within the article.
